# Exploring manzamine a: a promising anti-lung cancer agent from marine sponge *Haliclona* sp

**DOI:** 10.3389/fphar.2025.1525210

**Published:** 2025-02-25

**Authors:** Min Su, Jie Zhu, Luyuan Bai, Yu Cao, Shaohui Wang

**Affiliations:** ^1^ School of Pharmacy, Qingdao Medical College, Qingdao University, Qingdao, China; ^2^ Department of Scientific Research Management and Foreign Affairs, The Affiliated Hospital of Qingdao University, Qingdao, Shandong, China; ^3^ School of Basic Medicine, Qingdao Medical College, Qingdao University, Qingdao, China; ^4^ Clinical Trials Center, The Affiliated Hospital of Qingdao University, Qingdao, Shandong, China; ^5^ State Key Laboratory of Southwestern Chinese Medicine Resources, School of Ethnic Medicine, Chengdu University of Traditional Chinese Medicine, Chengdu, China

**Keywords:** manzamine a, lung cancer, network pharmacology, molecular docking, EGFR, SRC

## Abstract

Manzamine A (MA), a bioactive compound derived from the marine sponge *Haliclona* sp., shows considerable therapeutic potential, particularly in the treatment of various cancer types. Extracted with acetone and purified through chromatography, MA exhibits a bioavailability of 20.6% when administered orally in rats, underscoring its feasibility for therapeutic use. This compound disrupts key cellular mechanisms essential for cancer progression, including microtubule dynamics and DNA replication enzymes, demonstrating strong anti-proliferative effects against multiple cancer cell lines while sparing normal cells. Additionally, network pharmacology and molecular docking studies reveal MA’s interactions with important targets related to lung cancer progression, such as EGFR and SRC, bolstering its potential as a novel anti-lung cancer agent. Pathway analyses further indicate that MA influences critical signaling pathways involved in tumor growth and metastasis. Given the urgent need for effective treatments against drug-resistant cancers and the limited toxicity profile of MA, further exploration of its pharmacological benefits and mechanism could pave the way for new therapeutic strategies in lung cancer.

## Graphical Abstract

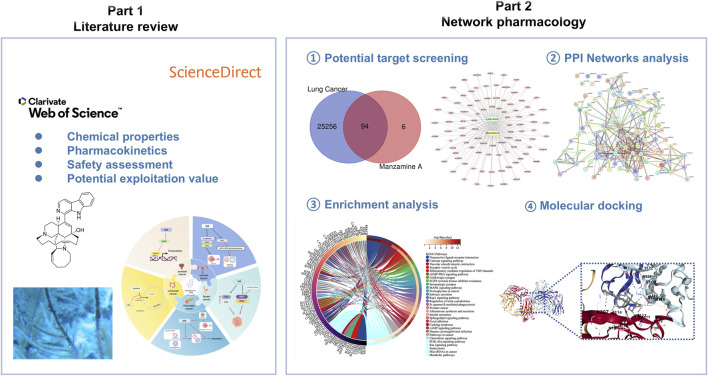

## 1 Introduction

Lung cancer ranks as the second most commonly diagnosed cancer globally and remains the leading cause of cancer-related deaths, surpassing breast, colorectal, and prostate cancers combined in 2020 (Siegel et al., 2023; Kratzer et al., 2024). By 2022, lung cancer had become the most diagnosed cancer worldwide, with nearly 2.5 million new cases, accounting for 12.4% of all cancer diagnoses (Bray et al., 2024). Projections suggest that the global cancer burden will double by 2050, further elevating lung cancer’s prominence (Nooreldeen and Bach, 2021). The two primary types of lung cancer are non-small cell lung cancer (NSCLC) and small cell lung cancer (SCLC), with NSCLC representing approximately 80% of cases (Nooreldeen and Bach, 2021; Liang et al., 2024). NSCLC encompasses subtypes such as adenocarcinoma, squamous cell carcinoma, and large cell carcinoma, while SCLC is characterized by its rapid growth and tendency to metastasize quickly, often associated with smoking (Risch and Plass, 2008; Warren and Cummings, 2013; Alam et al., 2021). Tobacco smoking is the primary risk factor for lung cancer, underscoring its significant impact on global health. The increasing incidence of lung cancer in developed nations is linked to long-term smoking and various environmental factors. The severity of lung cancer underscores the importance of targeted prevention strategies and early screening efforts to reduce mortality rates. Treatment for stage I or II NSCLC typically involves surgical removal and adjuvant therapy, while chemotherapy or radiation is commonly used for stage III or IV (Wathoni et al., 2022; Mathieu et al., 2023). However, conventional chemotherapy faces challenges such as non-specific targeting, low bioavailability, and the development of drug resistance, which can limit its effectiveness in cancer treatment (Holohan et al., 2013).

Numerous treatment strategies have been developed to combat lung cancer (Miyasaka et al., 2021; Ashrafi et al., 2022; Li et al., 2023; West and Kim, 2024). The treatment of lung cancer has entered the era of precision medicine, which includes chemotherapy, immunotherapy, targeted therapy, anti-angiogenesis therapy, and emerging treatment options. Chemotherapy remains a cornerstone for SCLC and certain subtypes of NSCLC, utilizing agents such as cisplatin, carboplatin, and paclitaxel (Barr et al., 2024). Immunotherapy, particularly PD-1/PD-L1 inhibitors (e.g., pembrolizumab, atezolizumab), is now considered first-line treatment for advanced NSCLC and SCLC (Roque et al., 2023). Targeted therapies that address mutations in EGFR, ALK, ROS1, and BRAF (e.g., osimertinib, crizotinib, dabrafenib) have significantly improved survival outcomes (Yu et al., 2024). Anti-angiogenesis agents, such as bevacizumab, when combined with chemotherapy and immunotherapy, can inhibit tumor growth (Siegel et al., 2023). Antibody-drug conjugates (ADCs) and nanomedicine delivery systems are at the forefront of therapeutic research (Ara and Hafeez, 2024). CAR-T therapy is currently under clinical investigation but remains experimental (La’ah and Chiou, 2024). These evolving strategies are enhancing survival rates, with ongoing research focused on optimizing personalized and combination treatments. However, lung cancer patients undergoing standard chemotherapy often face severe side effects due to the drug’s toxicity to normal tissues and the emergence of resistance. Traditional remedies have been used for centuries to treat a variety of diseases, including cancer (Banday et al., 2024). Marine natural products from animals, plants, and bacteria are a rich source for discovering and developing new, innovative treatments with unique mechanisms of action (Zhou et al., 2021). Sponges, which are immobile organisms, have developed complex defense mechanisms to protect themselves from predators, employing both physical and chemical strategies (Varijakzhan et al., 2021). These organisms produce a variety of bioactive compounds. Substances such as terpenes, alkaloids, and macrolides extracted from aquatic fungi, cyanobacteria, algae, sponges, and tunicates have shown diverse anti-cancer properties (Karthikeyan et al., 2022).

Manzamine A (MA), derived from sponges of the genera *Haliclona* sp., *Xestospongia* sp., and *Pellina* sp., features a pentacyclic core linked to a β-carboline alkaloid (Edrada et al., 1996; Watanabe et al., 1998). Studies indicate that MA exhibits anti-cancer effects against various types of cancer, including colorectal cancer, breast cancer, cervical cancer, pancreatic cancer, and prostate cancer (Kallifatidis et al., 2013; Lin et al., 2018; Karan et al., 2020; Wang et al., 2023; Karan et al., 2024). Although current treatments are effective for some patients, resistance remains a significant challenge to long-term efficacy. MA, with its multiple mechanisms of action, may help circumvent resistance issues associated with existing therapies. Specifically, MA could offer new avenues for lung cancer treatment by modulating several key pathways. The exploration of MA in comparison to traditional lung cancer therapies is a promising area of research. However, there is currently no literature supporting its specific role in lung cancer treatment or its comparison with conventional therapies. Thus, while MA’s anti-tumor effects have been validated in other cancers, its application in lung cancer warrants further research and clinical trials.

The innovative approach of network pharmacology, which integrates systems biology and medical data, has emerged as a powerful tool for predicting disease targets and gaining a comprehensive understanding of drug mechanisms (Guo et al., 2024; Qin et al., 2024; Xiang et al., 2024). When paired with molecular docking techniques, this approach enhances the ability to predict and validate the potential role of MA in treating lung cancer. Although research has demonstrated MA’s therapeutic effects on various conditions such as colorectal cancer, breast cancer, and cervical cancer, network pharmacology has yet to be employed to investigate its pharmacological activity specifically in lung cancer. Therefore, this review combines a literature survey with network pharmacology to examine MA’s chemical properties, pharmacokinetics, safety profile, and potential applications across different fields, with a particular emphasis on its pharmacological mechanisms and future research directions in lung cancer treatment. These findings provide valuable insights and guidance for the future development and application of MA in lung cancer therapy.

## 2 Chemical properties and pharmacokinetics of MA

MA (C_36_H_44_N_4_O, CAS: 104196-68-1) (Figure 2A) was first isolated in 1986 from the marine sponge *Haliclona* sp. collected near Cape Manzamo in Okinawa, Japan (Kubota et al., 2020). Featuring a complex structure with multiple rings and functional groups, including a fused and bridged tetra- or pentacyclic ring system linked to a β-carboline moiety, MA has shown potential in cancer treatment. These distinctive characteristics endow MA with various pharmacological activities, positioning it as a promising candidate for the treatment of cancer and malaria (Shilabin et al., 2008). The extraction of MA involves soaking the sponge in acetone to release its bioactive compounds. The process begins with the extraction of sponge samples using organic solvents like methanol or chloroform to yield a crude extract. This is followed by liquid-liquid extraction and chromatography techniques, such as silica gel column chromatography and reversed-phase liquid chromatography, to further separate and purify the compound, ultimately leading to the isolation of pure MA. These steps ensure the effective extraction of MA from the complex composition of the sponge while maintaining its purity (Sakai et al., 1987). Initially recognized for its potent antitumor properties, the potential medical applications of manzamine and its derivatives continue to be investigated (Pathak et al., 2015). Preliminary studies indicate that manzamine and its derivatives may be effective against a range of conditions, including malaria, herpes, HIV, and cancer, while also exhibiting antibacterial, antifungal, and anti-inflammatory effects (Hardy et al., 2022). As research progresses, it is becoming increasingly clear that this emerging compound could hold significant promise for the treatment of a diverse array of illnesses.

A solid understanding of the pharmacokinetics of MA is essential for determining appropriate dosages and studying potential drug interactions. In a pharmacokinetic study conducted in rats, an oral dose of 50 mg/kg of MA resulted in an oral bioavailability of approximately 20.6%, with a C_max_ of 1066 ± 177 ng/mL and a time to reach T_max_ of 10 ± 5 h (Yousaf et al., 2004). The pharmacokinetic analysis was performed using liquid chromatography-mass spectrometry (LC-MS), which provided precise measurements of MA concentrations over time. Non-compartmental pharmacokinetic analysis was employed to calculate key parameters such as half-life, volume of distribution, and clearance. MA demonstrated a relatively long half-life and low plasma clearance, indicating slow liver metabolism and good oral absorption, likely due to stable metabolic processes and favorable absorption characteristics enhanced by acid solubility and a high log *P* value (Yousaf et al., 2004). The study design also accounted for variability among subjects by including a diverse group of rats with variations in age, sex, and body mass. To evaluate differences in absorption, the coefficient of variation (CV%) was calculated for key pharmacokinetic parameters, including C_max_ and AUC. This analysis provided insights into inter-subject variability in MA’s pharmacokinetic properties, which is crucial for translating preclinical findings into clinical applications. These results underscore the potential of MA as a candidate for further preclinical evaluation and therapeutic development.

## 3 Safety assessment of MA

MA is an alkaline compound derived from sponges, known for its potential anti-cancer and anti-microbial properties. Despite its promising medical applications, research on its safety profile has been limited. *In vitro* studies have demonstrated that MA exhibits cytotoxic effects on various cancer cell lines, suggesting its potential to inhibit the growth of specific cancer cells. However, further assessment of its toxicity towards normal cells is essential to ensure selective targeting of cancer cells while minimizing harm to healthy tissues (Karan et al., 2020). Previous investigations by Hamann’s team have also shown that MA is effective against melanoma, prostate, and pancreatic cancer. Additionally, MA has demonstrated efficacy against the malaria parasite, leading to successful treatment outcomes in rodent models when administered alone (Ang et al., 2000; Rao et al., 2003). Moreover, an analog of MA, chloroquine, is currently being explored for its potential benefits in managing COVID-19, the disease caused by the novel coronavirus.

## 4 The potential exploitation value of MA

The substantial potential for the exploitation of manzamine A (MA) primarily resides in the medical field. Its unique structure and bioactivity profile suggest promising applications in combating cancer, viruses, inflammation, and malaria, particularly against drug-resistant strains of malaria and certain cancers in early research studies (Ashok et al., 2014). MA’s anticancer activity is a significant focus of research, with evidence indicating its ability to inhibit the growth of various cancer cell types, including colorectal, breast, and pancreatic cancers. This activity may be associated with its interference in microtubule dynamics, which are crucial for cancer cell growth and division. One of the earliest reports highlighting MA’s anti-cancer properties against leukemia dates back to the late 1990s, demonstrating potent anti-proliferative effects on multiple cancer cell lines, including leukemia cells. Several studies have investigated the cytotoxic effects of MA on different cell lines. At concentrations of 10–20 μM, osteoblast viability significantly decreased after 24–72 h. Additionally, Caspase 3/7 detection indicated that apoptosis in preosteoblasts and mature osteoblasts increased in a dose-dependent manner at 2.5 μM and 5 μM MA concentrations, with a decrease in apoptosis at 72 h in the 5 μM group, suggesting that peak apoptosis had occurred (Hardy et al., 2022). Another study evaluated the effects of MA on colorectal carcinoma cells using the MTS assay, revealing that MA reduced cell viability in HCT116, HT-29, and DLD-1 cells in a dose-dependent manner, with HCT116 exhibiting the highest sensitivity (IC_50_ = 4.5 μM). Colony formation assays further demonstrated that MA irreversibly inhibited HCT116 cell proliferation (Lin et al., 2018). The study also highlighted MA’s ability to inhibit DNA polymerase and topoisomerases, enzymes vital for DNA replication and cell division (Sakai et al., 1987).

Despite the significant promise shown by MA in inhibiting cancer cell proliferation, inducing apoptosis, and suppressing tumor growth, there is a notable lack of direct comparisons between MA and traditional chemotherapy agents such as paclitaxel, cisplatin, and doxorubicin. Research has indicated that MA diminishes the formation of individual pancreatic cancer cells, restricts cell movement, and enhances the susceptibility of AsPC-1 cells to TRAIL-induced apoptosis (Guzmán et al., 2011). Further studies have shown that MA can affect vacuolar ATPase in pancreatic cancer cells, impairing autophagy and potentially offering a beneficial treatment for cancer (Kallifatidis et al., 2013). The study showed that MA could potentially help CRC patients with poorly differentiated tumors by increasing E-cadherin expression, decreasing the activity of cancer-promoting proteins such as Snail and Slug, and preventing the movement of β-catenin into the cell nucleus (Lin et al., 2018). Molecular docking studies identified proteins and transcription factors regulated by CRC-related genes as potential drug targets, revealing that MA exhibited strong binding affinity for most target proteins, with an average affinity of −9.2 kcal/mol, and the TPX2-MA drug-ligand complex demonstrated stability (Islam et al., 2023). MA may thus help prevent the progression of colorectal cancer. In breast cancer, MA has shown notable activity and therapeutic effects. Wang et al. (2023) have reported that MA induces secretory autophagy in breast cancer cells by promoting autophagosome formation through inactivating the RIP1-mediated AKT/mTOR signaling pathway and inhibiting autophagosome degradation by reducing lysosomal acidity. This indicates that MA could potentially slow down or halt tumor growth. Furthermore, studies have indicated that MA is ten times more effective at inhibiting RSK1 compared to RSK2 *in vitro* and has been shown to suppress the expression of RSK1 and RSK2 proteins in human cervical cancer cells. RSK protein kinases are critical for cell growth, survival, and proliferation, operating downstream of the ras-ERK1/2 pathway. By inhibiting RSK1, MA disrupts this pathway, affecting the survival and proliferation of cervical cancer cells (Mayer et al., 2021). MA also disrupts the cell cycle in cervical cancer cells (SiHa and CaSki) at the G1/S phase and regulates key genes involved in the cell cycle, such as p21 and p53. It triggers apoptosis more effectively in HeLa cells and reduces Six1 levels, which are associated with cervical cancer development, underscoring its potential for both prevention and treatment (Karan et al., 2020). This research represents the first exploration of the anti-cancer effects of MA on prostate cancer, both *in vitro* and *in vivo*. The findings indicate that MA impedes the transcription of AR-FL and its variant AR-V7 by targeting E2F8, suggesting its potential as a therapeutic option for prostate cancer in both preclinical and clinical contexts (Karan et al., 2024).

**FIGURE 1:**
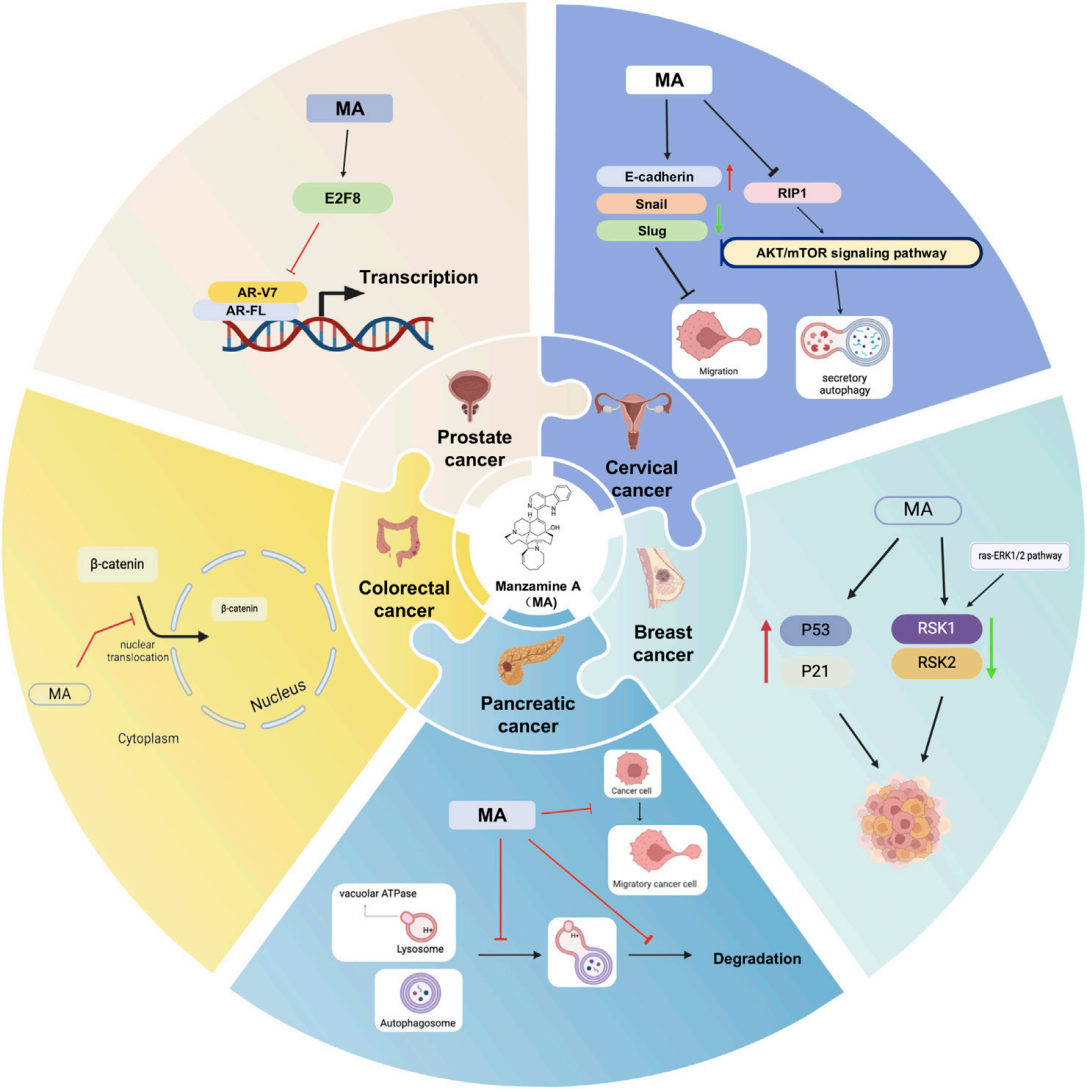
Potential mechanisms of MA for cancer. Black arrows indicate the order of linked responses. Red arrows show an increase in gene expression, component content, or specific indicators. Green arrows indicate a decrease in gene expression, component content, or the index of interest.

In conclusion, MA demonstrates significant promise in medicine, particularly for its anticancer properties. It disrupts critical cellular processes across various cancer types, including colorectal, breast, pancreatic, cervical, and prostate cancers, by targeting DNA replication, cell division, and apoptosis pathways. This highlights its potential for further preclinical and clinical development (Figure 1). Given MA’s efficacy in these cancers, researchers may consider investigating its effects on lung cancer. Such studies would involve detailed experiments to assess MA’s impact on lung cancer cells, including its influence on tumor growth, its capacity to induce apoptosis, and its role in lung cancer-related signaling pathways. Should these laboratory investigations reveal a potential therapeutic effect of MA on lung cancer, subsequent studies in animal models and preclinical trials could be conducted to evaluate its safety and effectiveness. Furthermore, the application of bioinformatics methods, such as network pharmacology and molecular docking, could be valuable in identifying specific lung cancer targets of MA and elucidating the molecular mechanisms behind its anticancer activity. These approaches would provide essential insights to inform future experimental and clinical research, even as the precise role of MA in lung cancer treatment remains to be fully clarified.

## 5 Network pharmacology analysis

Network pharmacology is an interdisciplinary approach designed to systematically clarify the multi-target and multi-pathway nature of drug actions by constructing complex networks that connect drugs, targets, and diseases (Zhao et al., 2023). This methodology incorporates advanced technologies such as bioinformatics, protein-protein interaction networks, molecular docking, and virtual screening to thoroughly analyze the interactions between drugs and biological systems, as well as to assess their potential side effects. By integrating diverse data from genomics, proteomics, and metabolomics, network pharmacology not only provides a comprehensive understanding of drug mechanisms but also supports the development of precision drug design and personalized therapeutic strategies (Hopkins, 2008).

### 5.1 Potential target screening

**FIGURE 2:**
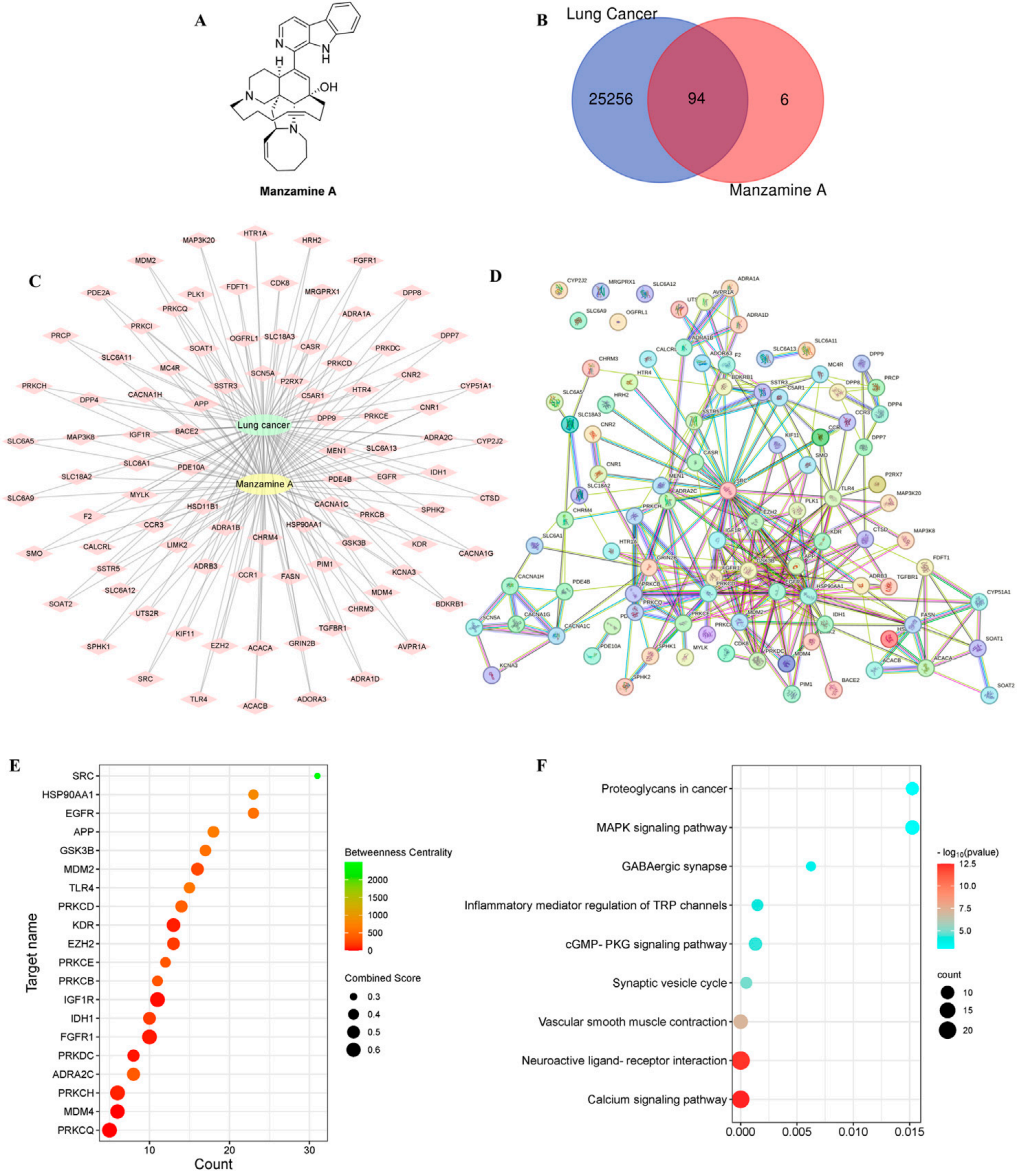
The exploration of potential molecular targets for treating lung cancer. **(A)** The molecular structure of MA. **(B)** The overlapping targets of MA and lung cancer using a Venn diagram. **(C)** The intricate interactions among the shared targets of MA and lung cancer through a diagram with green circles for lung cancer, yellow circles for MA, and diamond shapes for targets. The connections between MA, lung cancer, and shared targets are shown by lines. **(D)** The PPI network of shared targets, with proteins represented as points and associations indicated by lines. **(E)** The target centrality degree based on interaction counts, combination ability, and centrality degree, represented by changes in x-axis values, node colors, and node sizes. **(F)** A bubble diagram showing the KEGG pathway linked to lung cancer.

The Swiss Target Prediction online tool (http://www.swisstargetprediction.ch/) was used to identify the target genes of MA, yielding a total of 100 associated targets. To narrow these targets for lung cancer, we consulted the GeneCards database (https://www.genecards.org/, n = 25,951), the OMIM database (https://omim.org/, n = 526), and NCBI (https://www.ncbi.nlm.nih.gov/gene, n = 4,232), ultimately identifying 25,350 relevant targets after removing duplicates. The Venny 2.1 online tool (https://bioinfogp.cnb.csic.es/tools/venny/) was then employed to determine 94 common targets between lung cancer and MA (Figures 2B, C). To standardize these targets, the UniProt database was utilized to search for gene names within the “Human” species category.

### 5.2 Protein-protein interaction (PPI) networks analysis

The STRING online tool (https://cn.string-db.org/) was used to conduct a PPI analysis on the intersecting targets, with the species filter set to “*Homo sapiens*” and a minimum interaction score of 0.400. The resulting network visualized genes as nodes and their interactions as edges. Five target proteins with no interactions were excluded, yielding a PPI network consisting of 94 nodes (representing active proteins) and 268 edges (illustrating interactions among these active proteins and others) (Figure 2D). The top 20 targets were selected for enrichment mapping based on the number of interactions and binding capacity, noting an average node degree of 5.7 and an average local clustering coefficient of 0.569 (Figure 2E). Core targets were identified by assessing interaction counts, binding capacity, and centrality measures.

The PPI network diagram indicated that Epidermal Growth Factor Receptor (EGFR), Proto-oncogene Tyrosine-Protein Kinase Src (SRC), Heat Shock Protein HSP 90-alpha (HSP90AA1), Insulin-Like Growth Factor 1 Receptor Alpha Chain (IGF1R), and E3 Ubiquitin-Protein Ligase Mdm2 (MDM2) exhibited high scores. EGFR is vital for regulating epithelial tissues and is implicated in numerous cellular processes, including proliferation, survival, differentiation, and migration (Sabbah et al., 2020). Mutations in EGFR are found in 10%–40% of patients with NSCLC (Shigematsu and Gazdar, 2006). These mutations significantly influence the development and progression of lung cancer, and the immune response in EGFR mutation-driven cases is often weak, resulting in poor infiltration by anti-cancer immune effector cells and limited response to PD-1/PD-L1 therapies (Huang et al., 2022).

SRC, a non-receptor tyrosine kinase, is critical for cell survival in NSCLC tumorigenesis, interacting with cell surface growth factor receptors and intracellular signaling pathways. Elevated SRC levels in NSCLC have been associated with negative outcomes, increased cell movement, invasion, metastasis, drug resistance, and the activation of various tyrosine kinase receptors (He et al., 2013; Kuranami et al., 2015; Gu et al., 2017; Liu et al., 2019). The overexpression and hyperactivation of SRC have also been noted in various human cancers, leading to the upregulation of receptors such as EGFR and IGFR (Liu et al., 2015; Roskoski, 2015). SRC collaborates with factors like VEGF to promote tumor growth and metastasis, underscoring its significant role in tumor progression (Giaccone and Zucali, 2008). Targeting SRC and its signaling network is believed to improve treatment outcomes for NSCLC patients, with ongoing research exploring these interactions to develop more effective anti-cancer therapies.

HSP90AA1, also known as HSP90, is located on chromosome 14q32.2 and is a well-characterized protein composed of three domains: N-terminal, middle, and C-terminal (Gallegos Ruiz et al., 2008; Zuehlke et al., 2015). Its expression is significantly elevated in various cancers, including lung cancer (Birbo et al., 2021; Basset et al., 2023; Zhou et al., 2023). Research indicates that HSP90AA1 is associated with shorter overall survival in lung cancer patients, highlighting its relevance in tumor progression. Silencing HSP90AA1 has been shown to inhibit the AKT1 and ERK pathways, which are typically overactive in tumor tissues (Niu et al., 2021). Additionally, HSP90AA1 is downregulated in squamous cell carcinoma of the lung (a subtype of NSCLC), suggesting its potential role in the progression of this cancer type (Wang L. et al., 2020). Overall, HSP90AA1 functions as a critical molecular chaperone within signaling networks that support tumor growth and survival in lung cancer, making it a promising target for therapeutic intervention.

IGF1R is pivotal in tumor progression. A meta-analysis assessing the prognostic significance of IGF1R expression in NSCLC patients revealed that positive IGF1R expression correlates with poorer disease-free survival, as well as associations with smoking status and tumor size (Zhao et al., 2014). Disruption of IGF1R is common in various human cancers, leading to the activation of key pathways such as PI3K/AKT/mTOR and Ras/Raf/MAPK, which contribute to cancer cell proliferation, apoptosis, metastasis, and drug resistance (Ariga et al., 2000; Gallardo et al., 2012). IGF1R impacts survival, tumor development, and response to treatment, highlighting its potential as a therapeutic target in cancer treatment.

MDM2 is an enzyme that regulates the protein p53 by tagging it with ubiquitin (Gadepalli et al., 2014). In the context of NSCLC, the p38MAPK/MDM2/p53 signaling pathway has been well characterized. The activation of p38 MAPK can influence MDM2’s function, thereby affecting p53 levels and activity, which are critical for cell growth and survival. This pathway has garnered significant interest as a target for therapies aimed at modulating p53 and its regulatory mechanisms in cancer treatment (Zhang et al., 2021). MDM2 inhibitors have emerged as a promising area of research for lung cancer, focusing on disrupting the interaction between MDM2 and p53 to impede cancer progression (Konopleva et al., 2020).

In short, a PPI analysis utilizing the STRING tool revealed 94 active proteins and 268 interactions, highlighting key proteins associated with lung cancer, including EGFR, SRC, HSP90AA1, IGF1R, and MDM2. Both EGFR and SRC play vital roles in lung cancer progression, with EGFR mutations linked to diminished immune responses and SRC facilitating metastasis. HSP90AA1, a molecular chaperone, is associated with tumor survival, while IGF1R and MDM2 contribute to cancer proliferation and resistance through key signaling pathways. These insights emphasize the potential of targeting these proteins in the development of novel lung cancer therapies. To evaluate the therapeutic potential and underlying mechanisms of MA in lung cancer, both Gene Ontology (GO) enrichment analysis and Kyoto Encyclopedia of Genes and Genomes (KEGG) pathway analysis were conducted.

### 5.3 GO analysis and KEGG pathway analysis

**FIGURE 3:**
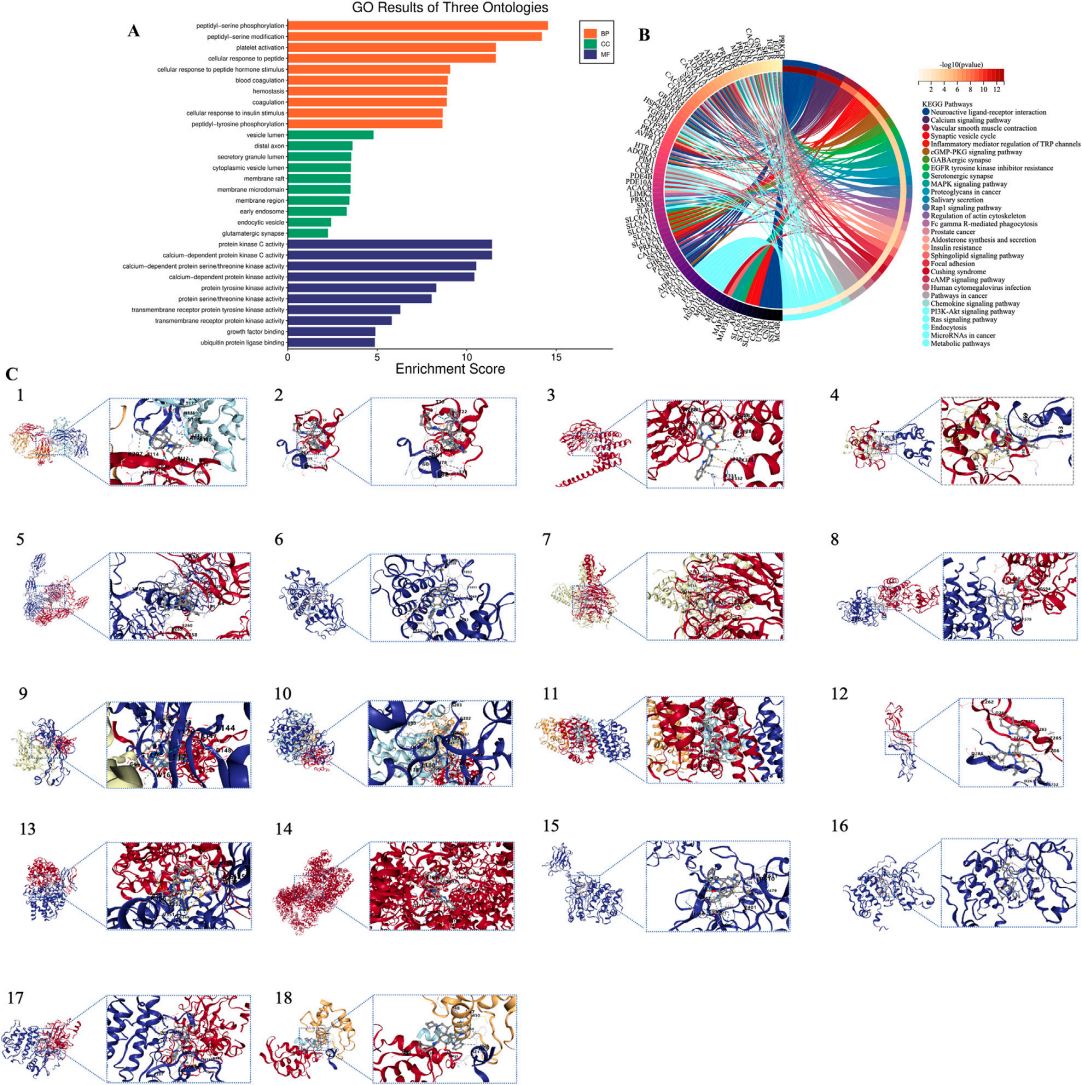
Bioinformatics analysis based on target genes of MA. **(A)** GO enrichment analysis. **(B)** KEGG pathway enrichment analysis. **(C)** Molecular docking results. 1. EGFR; 2. SRC; 3. HSP90AA1; 4. MDM2; 5. IGF1R; 6. KDR; 7. EZH2; 8. FGFR1; 9. APP; 10. GSK3B; 11. TLR4; 12. PRKCD; 13. IDH1; 14. PRKDC; 15. PRKCB; 16. PRKCH; 17. PRKCQ; 18. MDM4.

GO analysis and KEGG pathway analysis were conducted on the 64 core targets of MA, using a significance threshold of *P* < 0.05. The top enrichment results were classified into three categories: biological process (BP), molecular function (MF), and cellular component (CC), yielding 162 entries for BP, 34 for CC, and 58 for MF (Figure 3A). The categories of biological processes provide a comprehensive overview of the functional changes occurring within the body. To investigate the potential mechanisms by which MA may treat lung cancer, KEGG enrichment analysis was performed with the same significance threshold, identifying 206 signaling pathways. A selection of the top 30 key signaling pathways was illustrated in a loop graph (Figure 3B). Additionally, KEGG pathways specifically related to lung cancer were further enriched for a more detailed examination (Figure 2F).

In healthy cells, calcium (Ca^2+^) signals play a crucial role in regulating cell proliferation. However, cancer cells exhibit unique characteristics, including uncontrolled growth, enhanced spreading capabilities, and a propensity for metastasis. Unlike non-cancerous cells, the Ca^2+^ signaling pathways in cancer cells undergo reprogramming or dysregulation, which leads to alterations in their physiological properties (Zheng et al., 2021). Research indicates that disruptions in Ca^2+^ homeostasis contribute to tumor formation and progression (Wang W. J. et al., 2020). There is a growing recognition that changes in the expression or activation of specific proteins involved in Ca^2+^ regulation are linked to tumor development (Vandewalle et al., 1995; Gkika and Prevarskaya, 2011; Zhu et al., 2014). The balance of Ca^2+^ within cells is governed by a complex system of channels and transporters: (1) IP3 receptors and ryanodine receptors release Ca^2+^ from internal stores, such as the endoplasmic or sarcoplasmic reticulum; (2) Ca^2+^-ATPase pumps extrude Ca^2+^ from the cytosol into internal stores or out of the cell; (3) Various channels and transporters facilitate Ca^2+^ entry from the extracellular space, including voltage-gated Ca^2+^ channels, Transient receptor potential (TRP) channels, Ca^2+^ release-activated channels, Na^+^/Ca^2+^ exchangers, and purinergic receptors; (4) The mitochondrial Ca^2+^ uniporter regulates the uptake of Ca^2+^ into mitochondria (Cui et al., 2017). This intricate network of widely distributed Ca^2+^ channels, transporters, and pumps, along with their various activation processes, presents numerous potential targets for therapeutic intervention in cancer treatment.

TRP channels, a diverse group of ion channels found in various animal cell types, play a pivotal role in cellular functions such as sensation and signaling. These channels have garnered attention in the context of lung cancer for their influence on cancer cell physiology and tumor progression. TRP cation channels are essential for regulating the movement of positively charged ions like Na^+^ and Ca^2+^ across the cell membrane into the cytoplasm (Song and Yuan, 2010). Various types of TRP channels, including TRPV, TRPM, and TRPC, are expressed in lung tissues, each exhibiting unique characteristics that may differentially impact cancer cell behavior (Marini et al., 2023). TRP channels are integral to cancer development, as they influence cell proliferation, apoptosis, migration, and invasion. For example, TRPV6 has been shown to enhance cancer cell proliferation in NSCLC (Fan et al., 2014). Studies indicate that TRPV6 is highly expressed in SCLC tissues and cell lines compared to normal lung tissue (Lau et al., 2014). Additionally, TRPM7 receptors are critical in NSCLC progression, facilitating tumor cell migration, invasion, and metastasis. Specifically, TRPM7 promotes cancer progression by activating signaling pathways such as Hsp90α/uPA/MMP2, with its inhibition leading to decreased cancer cell motility and metastatic characteristics (Liu et al., 2018). Recent studies suggest that targeting the TRPM7/O-linked-β-N-acetylglucosaminylation (O-GlcNAcylation) axis may represent a promising therapeutic strategy to reduce lung cancer spread and enhance patient survival (Luanpitpong et al., 2020). Although TRP channels hold potential as therapeutic targets and biomarkers, further research is essential to fully realize their clinical applications.

The Rap1 signaling pathway in lung cancer plays a crucial role in regulating cell adhesion, migration, and survival, which are important factors for tumor progression and metastasis (Deregowska and Wnuk, 2021). In lung cancer, Rap1 can either enhance tumor growth by promoting cell survival and proliferation or hinder tumor development depending on the cellular context and tumor environment (Looi et al., 2020). Research has demonstrated that RAP1 protein levels are elevated in tumors from NSCLC, with higher concentrations observed in both the cytoplasm and nucleus compared to normal tissues, particularly in the cytoplasm (Xiao et al., 2017). Targeting the Rap1 signaling pathway could present novel therapeutic options in lung cancer. Inhibitors targeting Rap1 or its downstream effectors may be utilized to disrupt tumor cell adhesion, migration, or survival (Looi et al., 2020). Moreover, understanding the dual roles of Rap1 in tumor growth promotion or suppression could aid in developing targeted therapies considering the specific tumor context and patient genetic background.

For NSCLC patients with mutations in the EGFR who develop resistance to EGFR tyrosine kinase inhibitors (TKIs) through a process known as epithelial to mesenchymal transition (EMT), effective treatment strategies are urgently needed. Approximately 10%–15% of NSCLC patients have activating mutations in EGFR (Willmore-Payne et al., 2008). The standard initial treatment for these patients typically involves EGFR-TKIs, which are targeted therapies designed to inhibit the activity of the EGFR protein, often hyperactive due to mutations. By blocking EGFR activity, TKIs can help control the growth and spread of cancer cells in NSCLC patients with EGFR mutations (Zhang et al., 2023). EGFR-TKIs are preferred for initial treatment in this population, demonstrating superior efficacy compared to traditional chemotherapy in terms of response rates, progression-free survival (PFS), and quality of life (Su et al., 2020). A new treatment approach for specific types of lung cancer is currently being investigated by researchers, involving a combination of two drugs - amivantamab and lazertinib. Amivantamab targets two cancer growth-related proteins, while lazertinib fights EGFR gene mutations. These drugs are being evaluated together in phase I clinical trials known as CHRYSALIS and CHRYSALIS-2, being conducted in multiple countries. Preliminary results from these trials are promising, suggesting that this combination therapy may be effective (Bauml et al., 2021). The treatment of EGFR-mutant NSCLC has been transformed by EGFR-TKIs, leading to notable advancements in patient outcomes (Yoneda et al., 2019). Nevertheless, challenges such as resistance and side effects persist, necessitating ongoing research efforts to address these issues and further enhance drug efficacy.

The cGMP-PKG signaling pathway is recognized for its critical role in cancer progression, as demonstrated by various studies (Gong et al., 2019; Lv et al., 2020; Wang Z. et al., 2020; Chiang et al., 2024; Li et al., 2024). Current research is actively investigating the involvement of this pathway in lung cancer (Yang et al., 2024). By regulating apoptotic proteins, including those in the Bcl-2 family, the cGMP-PKG pathway can induce apoptosis in lung cancer cells (Wong et al., 2012). Investigations into the modulation of the cGMP-PKG pathway in lung cancer have primarily focused on its potential for novel therapeutic strategies.

In summary, network pharmacology analysis has identified that MA interacts with a substantial network of 64 core proteins linked to lung cancer, affecting key signaling pathways such as Ca^2+^ regulation, TRP channels, Rap1, EGFR, and cGMP-PKG. These pathways are crucial for essential cellular processes, including cell proliferation, apoptosis, migration, and metastasis, highlighting MA’s significant promise as a therapeutic agent for lung cancer. Despite these promising insights from pharmacological analyses regarding MA’s interaction with important pathways, including Ca^2+^ regulation, TRP channels, Rap1, EGFR, and cGMP-PKG, there remains a critical need for more concrete evidence. This can be achieved through rigorous *in vivo* and *in vitro* studies to confirm these findings and translate them into clinical applications. Particularly, the focus on targeting EGFR and addressing its resistance mechanisms offers a promising therapeutic avenue. Furthermore, exploring the roles of TRP channels and the Rap1 signaling pathway could lead to innovative interventions. Special emphasis should be placed on the cGMP-PKG pathway, which is pivotal in apoptosis, deeper investigation in this area may yield groundbreaking treatments in the battle against lung cancer. Overall, this expanded understanding calls for further exploration to unlock new avenues for lung cancer therapy.

## 6 Molecular docking

Molecular docking is a computational technique employed to predict the binding behavior between small molecules (drugs) and target proteins, elucidating binding sites, modes, and affinities (Burle et al., 2023). The process begins with obtaining the 3D structure of the target protein, typically through experimental methods such as X-ray crystallography or homology modeling. The ligand is then simulated to interact with the protein, and docking algorithms calculate the optimal binding mode and binding energy, with lower binding energies indicating stronger affinity. To validate these docking results, molecular dynamics simulations are conducted to assess the stability of the drug-protein complex, considering factors such as binding site stability, solvent effects, and conformational changes in the protein (Agu et al., 2023).

Based on findings from PPI and KEGG analyses, compound structural formulas were retrieved from the GeneCards website (https://www.genecards.org/) and saved in mol2 format. The 3D structures of receptor proteins were obtained from the PDB database, selected based on X-ray crystallography data and Angstrom (Å) values, and saved in PDB format. Blind docking analysis guided by cavity detection was performed using the online tool CB-DOCK2 (https://cadd.labshare.cn/cb-dock2/php/index.php), which predicts binding regions based on curvature and integrates with AutoDock Vina to explore interactions between MA and core targets. Data on binding energy, cavity volume, and center coordinates (x, y, z) were then collected to evaluate binding capacity. Molecules with docking energy below −5.0 kJ/mol indicate good binding ability, while those below −7.0 kJ/mol suggest excellent binding capability. Our molecular docking results revealed that the binding energies of EGFR, SRC, HSP90AA1, MDM2, IGF1R, KDR, EZH2, FGFR1, APP, GSK3B, TLR4, PRKCD, IDH1, PRKDC, PRKCB, PRKCH, PRKCQ, and MDM4 were all below −5.0 kJ/mol, indicating strong binding interactions. Notably, EGFR, MDM2, IGF1R, KDR, EZH2, GSK3B, TLR4, IDH1, PRKDC, and PRKCQ exhibited binding energies lower than −10.0 kJ/mol, suggesting exceptional binding strength and marking them as potential targets for further investigation. Among these, EZH2 displayed the most stable binding with MA, with a binding energy of −10 kJ/mol. EZH2 functions as a histone methyltransferase, specifically catalyzing histone H3 lysine 27 methylation. As a member of the PRC2 complex, EZH2 is accompanied by other components such as EED, SUZ12, and RpAp46/48 (Sun et al., 2022). EZH2 and MDM2 are critical in lung cancer; the activation of EZH2 accelerates cell growth, promoting rapid tumor progression and facilitating cancer cell dissemination to other organs by inducing EMT, thereby increasing cell mobility and invasiveness. Furthermore, elevated EZH2 levels in lung cancer are associated with reduced responsiveness to treatment, complicating disease management (Sun et al., 2014; Sun et al., 2016; Chen et al., 2020; Yang et al., 2021). Importantly, reducing EZH2 levels can slow tumor growth and enhance the effectiveness of anti-cancer drugs, making EZH2 inhibition a promising strategy in the fight against lung cancer (Gong et al., 2020). MDM2, another key player in lung cancer, inhibits the tumor-suppressive functions of p53, regulates cell cycle progression, prevents apoptosis, and facilitates cancer spread (Gadepalli et al., 2014). Targeting MDM2 presents a potential strategy to combat lung cancer by disrupting its interactions or inhibiting its activity. Researchers have also explored the role of circ-GSK3B, a circular RNA, in lung adenocarcinoma (LUAD), investigating its connection with glycogen synthase kinase 3 beta (GSK3B) and the Wnt/β-catenin signaling pathway. They found that circ-GSK3B acts as a tumor suppressor in LUAD by increasing and activating GSK3B (Zhu et al., 2022). The results of the molecular docking analyses can be found in Table 1 and Figure 3C.

**TABLE 1:** Molecular docking results between MA and core targets.

Protein	PDB ID	Binding Energy(kcal/mol)	Cavity volume (Å3)	Center (x, y, z)
EGFR	2XKN	−10.7	865	−33, 14, −29
SRC	2JYQ	−8.4	154	23,23,23
HSP90AA1	5NJX	−8.5	283	−26, 30, −21
MDM2	4LWV	−11.2	3106	−1, −25, −14
IGF1R	7XLC	−10.6	1199	115, 99, 127
KDR	3WZE	−10.4	213	19, 12, 32
EZH2	5WFC	−12.8	3684	5, 31, −17
FGFR1	4V05	−9.7	7654	57, −10, 11
APP	6HGA	−9.6	2885	−44, 116, −12
GSK3B	4J1R	−10.7	849	74, 50, 88
TLR4	5UC8	−10.9	2745	−8, −15, 11
PRKCD	3UEY	−8.6	285	−8, 8, 15
IDH1	5SVN	−11.8	5809	17, 21, −26
PRKDC	6ZFP	−10.3	5482	153, 115, 167
PRKCB	3PFQ	−9.1	2549	−43, 13, −17
PRKCH	3TXO	−8.4	1945	21, 16, 19
PRKCQ	5F9E	−11	2442	20, 70, −6
MDM4	6V4E	−9.6	4123	26, −8, 7

Overall, the molecular docking analysis indicated strong binding affinities between MA and several key proteins associated with lung cancer, including EGFR, MDM2, IGF1R, EZH2, and GSK3B, with binding energies below −10.0 kJ/mol, suggesting exceptional binding strength. Notably, EZH2 exhibited the most stable interaction with MA, underscoring its potential as a therapeutic target for lung cancer. Targeting EZH2, MDM2, and other identified proteins may inhibit tumor growth, enhance treatment responsiveness, and disrupt critical cancer pathways, offering promising strategies for the development of effective lung cancer therapies.

## 7 Conclusion

MA, a compound derived from certain marine sponges, particularly those of the genus Haliclona, shows promising potential in the treatment of lung cancer. These sponges produce a variety of bioactive substances as a defense mechanism against predators and competitors.

Research has demonstrated that MA possesses several pharmacological effects, including anti-inflammatory, antimicrobial, and anticancer properties. In the context of lung cancer, MA is gaining attention for its ability to inhibit cancer cell growth, induce programmed cell death, and impede the formation of new blood vessels that support tumor growth. However, existing therapies for resistant cancers, such as lung cancer, often face significant challenges, including the development of chemotherapy resistance, off-target effects, and limited efficacy in advanced stages. While chemotherapy and targeted therapies may be effective initially, they frequently lead to tumor recurrence due to the emergence of resistant cell populations, highlighting the need for new treatment options. One notable aspect of MA is its broad mechanism of action, which targets multiple molecular pathways involved in cancer development and progression, including key enzymes and regulators of the cell cycle. For example, MA inhibits phosphodiesterase and protein kinases that play critical roles in cell signaling and proliferation. This multifaceted action provides an advantage over conventional therapies, which often focus on a single target and risk inducing resistance. By addressing multiple proteins involved in cancer progression, MA may help to prevent or delay the onset of resistance.

Moreover, MA exhibits minimal toxicity towards healthy cells, which is crucial in cancer treatment to avoid damaging normal tissues. This reduced toxicity is a significant advantage, as many conventional chemotherapy agents cause substantial side effects due to their non-selective effects on both cancerous and healthy cells. Although these findings are promising, further studies are necessary to fully understand the benefits of using MA for lung cancer treatment. It is also essential to investigate the potential risks associated with long-term use, including the possibility of tumor cells developing resistance to MA. Clinical trials are vital to assess the safety and efficacy of MA in human patients, as well as to determine optimal dosing and potential combination therapies. Such trials should also evaluate the long-term safety of MA, as resistance to multi-targeted agents could still present challenges, particularly in advanced cancer stages.

MA exerts its anticancer effects by targeting key proteins such as EGFR, SRC, HSP90AA1, MDM2, and IGF1R, which are often overexpressed or mutated in resistant cancer cells. A multi-targeted approach like MA could provide more durable treatment outcomes compared to therapies that target a single pathway. The significance of the pharmacological mechanisms and potential research directions for MA in lung cancer treatment cannot be overstated. Continued research will clarify the specific mechanisms of action of MA, facilitating its clinical application.

In conclusion, this study provides valuable insights and guidance for the development and utilization of MA in lung cancer therapy. MA represents a promising opportunity for innovative and effective lung cancer treatments. Its unique characteristics, multi-targeted mechanism of action, and low toxicity profile position it as a viable candidate to address the challenges faced by current cancer therapies, including drug resistance and toxicity. Nonetheless, ongoing research and clinical validation are crucial to confirming its potential and addressing the challenges of resistance and long-term safety.

## References

[B1] Agu P. C. Afiukwa C. A. Orji O. U. Ezeh E. M. Ofoke I. H. Ogbu C. O. (2023). Molecular docking as a tool for the discovery of molecular targets of nutraceuticals in diseases management. Sci. Rep. 13, 13398. 10.1038/s41598-023-40160-2 37592012 PMC10435576

[B2] Alam A. Ansari M. A. Badrealam K. F. Pathak S. (2021). Molecular approaches to lung cancer prevention. Future Oncol. 17, 1793–1810. 10.2217/fon-2020-0789 33653087

[B3] Ang K. K. Holmes M. J. Higa T. Hamann M. T. Kara U. A. (2000). *In vivo* antimalarial activity of the beta-carboline alkaloid manzamine A. Antimicrob. Agents Chemother. 44, 1645–1649. 10.1128/aac.44.6.1645-1649.2000 10817722 PMC89926

[B4] Ara N. Hafeez A. (2024). Nanocarrier-Mediated drug delivery via Inhalational route for lung cancer therapy: a systematic and updated review. AAPS PharmSciTech 25, 47. 10.1208/s12249-024-02758-1 38424367

[B5] Ariga M. Nedachi T. Akahori M. Sakamoto H. Ito Y. Hakuno F. (2000). Signalling pathways of insulin-like growth factor-I that are augmented by cAMP in FRTL-5 cells. Biochem. J. 348 (Pt 2), 409–416. 10.1042/bj3480409 10816436 PMC1221080

[B6] Ashok P. Ganguly S. Murugesan S. (2014). Manzamine alkaloids: isolation, cytotoxicity, antimalarial activity and SAR studies. Drug Discov. Today 19, 1781–1791. 10.1016/j.drudis.2014.06.010 24953707

[B7] Ashrafi A. Akter Z. Modareszadeh P. Modareszadeh P. Berisha E. Alemi P. S. (2022). Current landscape of therapeutic resistance in lung cancer and promising strategies to overcome resistance. Cancers (Basel) 14, 4562. 10.3390/cancers14194562 36230484 PMC9558974

[B8] Banday A. H. Azha N. U. Farooq R. Sheikh S. A. Ganie M. A. Parray M. N. (2024). Exploring the potential of marine natural products in drug development: a comprehensive review. Phytochem. Lett. 59, 124–135. 10.1016/j.phytol.2024.01.001

[B9] Barr T. Ma S. Li Z. Yu J. (2024). Recent advances and remaining challenges in lung cancer therapy. Chin. Med. J. Engl. 137, 533–546. 10.1097/cm9.0000000000002991 38321811 PMC10932530

[B10] Basset C. A. Conway De Macario E. Leone L. G. Macario A. J. L. Leone A. (2023). The chaperone system in cancer therapies: Hsp90. J. Mol. Histol. 54, 105–118. 10.1007/s10735-023-10119-8 36933095 PMC10079721

[B11] Bauml J. Cho B. C. Park K. Lee K. H. Cho E. K. Kim D.-W. (2021). Amivantamab in combination with lazertinib for the treatment of osimertinib-relapsed, chemotherapy-naïve EGFR mutant (EGFRm) non-small cell lung cancer (NSCLC) and potential biomarkers for response. J. Clin. Oncol. 39, 9006. 10.1200/JCO.2021.39.15_suppl.9006

[B12] Birbo B. Madu E. E. Madu C. O. Jain A. Lu Y. (2021). Role of HSP90 in cancer. Int. J. Mol. Sci. 22, 10317. 10.3390/ijms221910317 34638658 PMC8508648

[B13] Bray F. Laversanne M. Sung H. Ferlay J. Siegel R. L. Soerjomataram I. (2024). Global cancer statistics 2022: GLOBOCAN estimates of incidence and mortality worldwide for 36 cancers in 185 countries. CA Cancer J. Clin. 74, 229–263. 10.3322/caac.21834 38572751

[B14] Burle S. Gupta K. Jibhkate Y. Hemke A. Umekar M. (2023). Insights into molecular docking: a comprehensive view. Int. J. Pharm. Chem. Analysis 10, 175–184. 10.18231/j.ijpca.2023.030

[B15] Chen L. Zhuo H. Z. Wu J. Y. Lin L. Y. Huang Z. L. Lu J. X. (2020). MiR-92b inhibits proliferation and invasion of lung cancer by targeting EZH2. Eur. Rev. Med. Pharmacol. Sci. 24, 3166–3173. 10.26355/eurrev_202003_20683 32271434

[B16] Chiang J. Y. Wei S. T. Chang H. J. Chen D. C. Wang H. L. Lei F. J. (2024). ABCC4 suppresses glioblastoma progression and recurrence by restraining cGMP-PKG signalling. Br. J. Cancer 130, 1324–1336. 10.1038/s41416-024-02581-2 38347095 PMC11014854

[B17] Cui C. Merritt R. Fu L. Pan Z. (2017). Targeting calcium signaling in cancer therapy. Acta Pharm. Sin. B 7, 3–17. 10.1016/j.apsb.2016.11.001 28119804 PMC5237760

[B18] Deregowska A. Wnuk M. (2021). RAP1/TERF2IP-A multifunctional player in cancer development. Cancers (Basel) 13, 5970. 10.3390/cancers13235970 34885080 PMC8657031

[B19] Edrada R. A. Proksch P. Wray V. Witte L. Müller W. E. Van Soest R. W. (1996). Four new bioactive manzamine-type alkaloids from the Philippine marine sponge Xestospongia ashmorica. J. Nat. Prod. 59, 1056–1060. 10.1021/np9604083 8946747

[B20] Fan H. Shen Y. X. Yuan Y. F. (2014). Expression and prognostic roles of TRPV5 and TRPV6 in non-small cell lung cancer after curative resection. Asian Pac J. Cancer Prev. 15, 2559–2563. 10.7314/apjcp.2014.15.6.2559 24761864

[B21] Gadepalli V. S. Deb S. P. Deb S. Rao R. R. (2014). Lung cancer stem cells, p53 mutations and MDM2. Subcell. Biochem. 85, 359–370. 10.1007/978-94-017-9211-0_19 25201204

[B22] Gallardo A. Lerma E. Escuin D. Tibau A. Muñoz J. Ojeda B. (2012). Increased signalling of EGFR and IGF1R, and deregulation of PTEN/PI3K/Akt pathway are related with trastuzumab resistance in HER2 breast carcinomas. Br. J. Cancer 106, 1367–1373. 10.1038/bjc.2012.85 22454081 PMC3326683

[B23] Gallegos Ruiz M. I. Floor K. Roepman P. Rodriguez J. A. Meijer G. A. Mooi W. J. (2008). Integration of gene dosage and gene expression in non-small cell lung cancer, identification of HSP90 as potential target. PLoS One 3, e0001722. 10.1371/journal.pone.0001722 18320023 PMC2254495

[B24] Giaccone G. Zucali P. A. (2008). Src as a potential therapeutic target in non-small-cell lung cancer. Ann. Oncol. 19, 1219–1223. 10.1093/annonc/mdn048 18388349

[B25] Gkika D. Prevarskaya N. (2011). TRP channels in prostate cancer: the good, the bad and the ugly? Asian J. Androl. 13, 673–676. 10.1038/aja.2011.18 21623387 PMC3739589

[B26] Gong H. Li Y. Yuan Y. Li W. Zhang H. Zhang Z. (2020). EZH2 inhibitors reverse resistance to gefitinib in primary EGFR wild-type lung cancer cells. BMC Cancer 20, 1189. 10.1186/s12885-020-07667-7 33276757 PMC7716470

[B27] Gong L. Lei Y. Tan X. Dong Y. Luo Z. Zhang D. (2019). Propranolol selectively inhibits cervical cancer cell growth by suppressing the cGMP/PKG pathway. Biomed. Pharmacother. 111, 1243–1248. 10.1016/j.biopha.2019.01.027 30841438

[B28] Gu Z. Fang X. Li C. Chen C. Liang G. Zheng X. (2017). Increased PTPRA expression leads to poor prognosis through c-Src activation and G1 phase progression in squamous cell lung cancer. Int. J. Oncol. 51, 489–497. 10.3892/ijo.2017.4055 28656243 PMC5505127

[B29] Guo S. Wu C. Liu X. Meng X. Zhang Y. Wang S. (2024). Deciphering the healing power of Swertia Chirayita: A potential treatment for acute liver injury. Arabian J. Chem. 17, 105930. 10.1016/j.arabjc.2024.105930

[B30] Guzmán E. A. Johnson J. D. Linley P. A. Gunasekera S. E. Wright A. E. (2011). A novel activity from an old compound: Manzamine A reduces the metastatic potential of AsPC-1 pancreatic cancer cells and sensitizes them to TRAIL-induced apoptosis. Invest New Drugs 29, 777–785. 10.1007/s10637-010-9422-6 20352293 PMC3085053

[B31] Hardy S. Choo Y. M. Hamann M. Cray J. (2022). Manzamine-A alters In Vitro calvarial osteoblast function. Mar. Drugs 20, 647. 10.3390/md20100647 36286470 PMC9604769

[B32] He P. Wu W. Wang H. Liao K. Zhang W. Xiong G. (2013). Co-expression of Rho guanine nucleotide exchange factor 5 and Src associates with poor prognosis of patients with resected non-small cell lung cancer. Oncol. Rep. 30, 2864–2870. 10.3892/or.2013.2797 24126923

[B33] Holohan C. Van Schaeybroeck S. Longley D. B. Johnston P. G. (2013). Cancer drug resistance: an evolving paradigm. Nat. Rev. Cancer 13, 714–726. 10.1038/nrc3599 24060863

[B34] Hopkins A. L. (2008). Network pharmacology: the next paradigm in drug discovery. Nat. Chem. Biol. 4, 682–690. 10.1038/nchembio.118 18936753

[B35] Huang M. Xiong D. Pan J. Zhang Q. Sei S. Shoemaker R. H. (2022). Targeting glutamine metabolism to enhance immunoprevention of EGFR-driven lung cancer. Adv. Sci. (Weinh) 9, e2105885. 10.1002/advs.202105885 35861366 PMC9475521

[B36] Islam M. A. Hossen M. B. Horaira M. A. Hossen M. A. Kibria M. K. Reza M. S. (2023). Exploring core genes by comparative transcriptomics analysis for early diagnosis, prognosis, and therapies of colorectal cancer. Cancers (Basel) 15, 1369. 10.3390/cancers15051369 36900162 PMC10000172

[B37] Kallifatidis G. Hoepfner D. Jaeg T. Guzmán E. A. Wright A. E. (2013). The marine natural product manzamine A targets vacuolar ATPases and inhibits autophagy in pancreatic cancer cells. Mar. Drugs 11, 3500–3516. 10.3390/md11093500 24048269 PMC3806460

[B38] Karan D. Dubey S. Gunewardena S. Iczkowski K. A. Singh M. Liu P. (2024). Manzamine A reduces androgen receptor transcription and synthesis by blocking E2F8-DNA interactions and effectively inhibits prostate tumor growth in mice. Mol. Oncol. 18, 1966–1979. 10.1002/1878-0261.13637 38605607 PMC11306517

[B39] Karan D. Dubey S. Pirisi L. Nagel A. Pina I. Choo Y. M. (2020). The marine natural product manzamine A inhibits cervical cancer by targeting the SIX1 protein. J. Nat. Prod. 83, 286–295. 10.1021/acs.jnatprod.9b00577 32022559 PMC7161578

[B40] Karthikeyan A. Joseph A. Nair B. G. (2022). Promising bioactive compounds from the marine environment and their potential effects on various diseases. J. Genet. Eng. Biotechnol. 20, 14. 10.1186/s43141-021-00290-4 35080679 PMC8790952

[B41] Konopleva M. Martinelli G. Daver N. Papayannidis C. Wei A. Higgins B. (2020). MDM2 inhibition: an important step forward in cancer therapy. Leukemia 34, 2858–2874. 10.1038/s41375-020-0949-z 32651541

[B42] Kratzer T. B. Bandi P. Freedman N. D. Smith R. A. Travis W. D. Jemal A. (2024). Lung cancer statistics, 2023. Cancer 130, 1330–1348. 10.1002/cncr.35128 38279776

[B43] Kubota T. Kurimoto S. I. Kobayashi J. (2020). The manzamine alkaloids. Alkaloids Chem. Biol. 84, 1–124. 10.1016/bs.alkal.2020.03.001 32416951

[B44] Kuranami S. Yokobori T. Mogi A. Altan B. Yajima T. Onozato R. (2015). Src kinase-associated phosphoprotein2 expression is associated with poor prognosis in non-small cell lung cancer. Anticancer Res. 35, 2411–2415. 25862907

[B45] La’ah A. Chiou S.-H. (2024). Cutting-edge therapies for lung cancer. Cells 13, 436. 10.3390/cells13050436 38474400 PMC10930724

[B46] Lau J. K. Brown K. C. Dom A. M. Witte T. R. Thornhill B. A. Crabtree C. M. (2014). Capsaicin induces apoptosis in human small cell lung cancer via the TRPV6 receptor and the calpain pathway. Apoptosis 19, 1190–1201. 10.1007/s10495-014-1007-y 24878626 PMC4072851

[B47] Li X. Liu B. Wang S. Dong Q. Li J. (2024). EDNRB inhibits the growth and migration of prostate cancer cells by activating the cGMP-PKG pathway. Open Med. (Wars) 19, 20230875. 10.1515/med-2023-0875 38205153 PMC10775416

[B48] Li Y. Yan B. He S. (2023). Advances and challenges in the treatment of lung cancer. Biomed. Pharmacother. 169, 115891. 10.1016/j.biopha.2023.115891 37979378

[B49] Liang S. Cao X. Wang Y. Leng P. Wen X. Xie G. (2024). Metabolomics analysis and diagnosis of lung cancer: insights from diverse sample types. Int. J. Med. Sci. 21, 234–252. 10.7150/ijms.85704 38169594 PMC10758149

[B50] Lin L. C. Kuo T. T. Chang H. Y. Liu W. S. Hsia S. M. Huang T. C. (2018). Manzamine A exerts anticancer activity against human colorectal cancer cells. Mar. Drugs 16, 252. 10.3390/md16080252 30060617 PMC6117705

[B51] Liu K. Xu S. H. Chen Z. Zeng Q. X. Li Z. J. Chen Z. M. (2018). TRPM7 overexpression enhances the cancer stem cell-like and metastatic phenotypes of lung cancer through modulation of the Hsp90α/uPA/MMP2 signaling pathway. BMC Cancer 18, 1167. 10.1186/s12885-018-5050-x 30477473 PMC6258145

[B52] Liu W. Kovacevic Z. Peng Z. Jin R. Wang P. Yue F. (2015). The molecular effect of metastasis suppressors on Src signaling and tumorigenesis: new therapeutic targets. Oncotarget 6, 35522–35541. 10.18632/oncotarget.5849 26431493 PMC4742122

[B53] Liu W. Liang Y. Chan Q. Jiang L. Dong J. (2019). CX3CL1 promotes lung cancer cell migration and invasion via the Src/focal adhesion kinase signaling pathway. Oncol. Rep. 41, 1911–1917. 10.3892/or.2019.6957 30628679

[B54] Looi C. K. Hii L. W. Ngai S. C. Leong C. O. Mai C. W. (2020). The role of ras-associated protein 1 (Rap1) in cancer: bad actor or good player? Biomedicines 8, 334. 10.3390/biomedicines8090334 32906721 PMC7555474

[B55] Luanpitpong S. Rodboon N. Samart P. Vinayanuwattikun C. Klamkhlai S. Chanvorachote P. (2020). A novel TRPM7/O-GlcNAc axis mediates tumour cell motility and metastasis by stabilising c-Myc and caveolin-1 in lung carcinoma. Br. J. Cancer 123, 1289–1301. 10.1038/s41416-020-0991-7 32684624 PMC7555538

[B56] Lv Y. Wang X. Li X. Xu G. Bai Y. Wu J. (2020). Nucleotide *de novo* synthesis increases breast cancer stemness and metastasis via cGMP-PKG-MAPK signaling pathway. PLoS Biol. 18, e3000872. 10.1371/journal.pbio.3000872 33186350 PMC7688141

[B57] Marini M. Titiz M. Souza Monteiro De Araújo D. Geppetti P. Nassini R. De Logu F. (2023). TRP channels in cancer: signaling mechanisms and translational approaches. Biomolecules 13, 1557. 10.3390/biom13101557 37892239 PMC10605459

[B58] Mathieu L. N. Larkins E. Sinha A. K. Mishra-Kalyani P. S. Jafri S. Kalavar S. (2023). FDA approval summary: atezolizumab as adjuvant treatment following surgical resection and platinum-based chemotherapy for stage II to IIIA NSCLC. Clin. Cancer Res. 29, 2973–2978. 10.1158/1078-0432.ccr-22-3699 36951523 PMC10440223

[B59] Mayer A. M. S. Hall M. L. Lach J. Clifford J. Chandrasena K. Canton C. (2021). RSK1 vs. RSK2 inhibitory activity of the marine β-carboline alkaloid manzamine A: a biochemical, cervical cancer protein expression, and computational study. Mar. Drugs 19, 506. 10.3390/md19090506 34564169 PMC8467814

[B60] Miyasaka Y. Sato H. Okano N. Kubo N. Kawamura H. Ohno T. (2021). A promising treatment strategy for lung cancer: a combination of radiotherapy and immunotherapy. Cancers (Basel) 14, 203. 10.3390/cancers14010203 35008367 PMC8750493

[B61] Niu M. Zhang B. Li L. Su Z. Pu W. Zhao C. (2021). Targeting HSP90 inhibits proliferation and induces apoptosis through AKT1/ERK pathway in lung cancer. Front. Pharmacol. 12, 724192. 10.3389/fphar.2021.724192 35095481 PMC8795737

[B62] Nooreldeen R. Bach H. (2021). Current and future development in lung cancer diagnosis. Int. J. Mol. Sci. 22, 8661. 10.3390/ijms22168661 34445366 PMC8395394

[B63] Pathak R. B. Dobson B. C. Ghosh N. Ageel K. A. Alshawish M. R. Saruengkhanphasit R. (2015). Synthesis of the tricyclic core of manzamine A. Org. Biomol. Chem. 13, 3331–3340. 10.1039/c4ob02582b 25647730

[B64] Qin J. Xiang G. Gao H. Meng X. Wang S. Zhang Y. (2024). Determination of the pharmacodynamic substances and mechanism of Shiwuwei Saierdou Pills against cholestatic hepatitis through chemical profile identification and network pharmacology analysis. Arabian J. Chem. 17, 105504. 10.1016/j.arabjc.2023.105504

[B65] Rao K. V. Santarsiero B. D. Mesecar A. D. Schinazi R. F. Tekwani B. L. Hamann M. T. (2003). New manzamine alkaloids with activity against infectious and tropical parasitic diseases from an Indonesian sponge. J. Nat. Prod. 66, 823–828. 10.1021/np020592u 12828469 PMC4969047

[B66] Risch A. Plass C. (2008). Lung cancer epigenetics and genetics. Int. J. Cancer 123, 1–7. 10.1002/ijc.23605 18425819

[B67] Roque K. Ruiz R. Mas L. Pozza D. H. Vancini M. Silva Júnior J. A. (2023). Update in immunotherapy for advanced non-small cell lung cancer: Optimizing treatment sequencing and identifying the best choices. Cancers (Basel) 15, 4547. 10.3390/cancers15184547 37760516 PMC10526179

[B68] Roskoski R. Jr. (2015). Src protein-tyrosine kinase structure, mechanism, and small molecule inhibitors. Pharmacol. Res. 94, 9–25. 10.1016/j.phrs.2015.01.003 25662515

[B69] Sabbah D. A. Hajjo R. Sweidan K. (2020). Review on epidermal growth factor receptor (EGFR) structure, signaling pathways, interactions, and recent updates of EGFR inhibitors. Curr. Top. Med. Chem. 20, 815–834. 10.2174/1568026620666200303123102 32124699

[B70] Sakai R. Higa T. Jefford C. W. Bernardinelli G. (1987). ChemInform abstract: manzamine A, a novel antitumor alkaloid from a sponge. ChemInform 18. 10.1002/chin.198706071

[B71] Shigematsu H. Gazdar A. F. (2006). Somatic mutations of epidermal growth factor receptor signaling pathway in lung cancers. Int. J. Cancer 118, 257–262. 10.1002/ijc.21496 16231326

[B72] Shilabin A. G. Kasanah N. Tekwani B. L. Hamann M. T. (2008). Kinetic studies and bioactivity of potential manzamine prodrugs. J. Nat. Prod. 71, 1218–1221. 10.1021/np800163u 18598080 PMC4918903

[B73] Siegel R. L. Miller K. D. Wagle N. S. Jemal A. (2023). Cancer statistics, 2023. CA Cancer J. Clin. 73, 17–48. 10.3322/caac.21763 36633525

[B74] Song M. Y. Yuan J. X. (2010). Introduction to TRP channels: structure, function, and regulation. Adv. Exp. Med. Biol. 661, 99–108. 10.1007/978-1-60761-500-2_6 20204725

[B75] Su V. Y. Yang K. Y. Huang T. Y. Hsu C. C. Chen Y. M. Yen J. C. (2020). The efficacy of first-line tyrosine kinase inhibitors combined with co-medications in Asian patients with EGFR mutation non-small cell lung cancer. Sci. Rep. 10, 14965. 10.1038/s41598-020-71583-w 32917914 PMC7486374

[B76] Sun C. C. Li S. J. Li G. Hua R. X. Zhou X. H. Li D. J. (2016). Long intergenic noncoding RNA 00511 acts as an oncogene in non-small-cell lung cancer by binding to EZH2 and suppressing p57. Mol. Ther. Nucleic Acids 5, e385. 10.1038/mtna.2016.94 27845772 PMC5155326

[B77] Sun M. Liu X. H. Lu K. H. Nie F. Q. Xia R. Kong R. (2014). EZH2-mediated epigenetic suppression of long noncoding RNA SPRY4-IT1 promotes NSCLC cell proliferation and metastasis by affecting the epithelial-mesenchymal transition. Cell Death Dis. 5, e1298. 10.1038/cddis.2014.256 24967960 PMC4611729

[B78] Sun S. Yu F. Xu D. Zheng H. Li M. (2022). EZH2, a prominent orchestrator of genetic and epigenetic regulation of solid tumor microenvironment and immunotherapy. Biochim. Biophys. Acta Rev. Cancer 1877, 188700. 10.1016/j.bbcan.2022.188700 35217116

[B79] Vandewalle B. Hornez L. Wattez N. Revillion F. Lefebvre J. (1995). Vitamin-D3 derivatives and breast-tumor cell growth: effect on intracellular calcium and apoptosis. Int. J. Cancer 61, 806–811. 10.1002/ijc.2910610611 7790115

[B80] Varijakzhan D. Loh J. Y. Yap W. S. Yusoff K. Seboussi R. Lim S. E. (2021). Bioactive compounds from marine sponges: Fundamentals and applications. Mar. Drugs 19, 246. 10.3390/md19050246 33925365 PMC8146879

[B81] Wang L. Zhao H. Zhang L. Luo H. Chen Q. Zuo X. (2020a). HSP90AA1, ADRB2, TBL1XR1 and HSPB1 are chronic obstructive pulmonary disease-related genes that facilitate squamous cell lung cancer progression. Oncol. Lett. 19, 2115–2122. 10.3892/ol.2020.11318 32194709 PMC7039115

[B82] Wang W. J. Mao L. F. Lai H. L. Wang Y. W. Jiang Z. B. Li W. (2020b). Dolutegravir derivative inhibits proliferation and induces apoptosis of non-small cell lung cancer cells via calcium signaling pathway. Pharmacol. Res. 161, 105129. 10.1016/j.phrs.2020.105129 32783976

[B83] Wang X. Liu Y. Qin H. Qi G. Chen X. Lyu Y. (2023). RIP1 mediates manzamine-A-induced secretory autophagy in breast cancer. Mar. Drugs 21, 151. 10.3390/md21030151 36976201 PMC10051755

[B84] Wang Z. Zhang C. Chang J. Tian X. Zhu C. Xu W. (2020c). LncRNA EMX2OS, regulated by TCF12, interacts with FUS to regulate the proliferation, migration and invasion of prostate cancer cells through the cGMP-PKG signaling pathway. Onco Targets Ther. 13, 7045–7056. 10.2147/ott.s243552 32801740 PMC7398891

[B85] Warren G. W. Cummings K. M. (2013). Tobacco and lung cancer: risks, trends, and outcomes in patients with cancer. Am. Soc. Clin. Oncol. Educ. Book **.** 359–364. 10.14694/EdBook_AM.2013.33.359 23714547

[B86] Watanabe D. Tsuda M. Kobayashi J. (1998). Three new manzamine congeners from amphimedon sponge. J. Nat. Prod. 61, 689–692. 10.1021/np970564p 9599281

[B87] Wathoni N. Puluhulawa L. E. Joni I. M. Muchtaridi M. Mohammed A. F. A. Elamin K. M. (2022). Monoclonal antibody as a targeting mediator for nanoparticle targeted delivery system for lung cancer. Drug Deliv. 29, 2959–2970. 10.1080/10717544.2022.2120566 36085575 PMC9467540

[B88] West H. J. Kim J. Y. (2024). Rapid advances in resectable non-small cell lung cancer: a narrative review. JAMA Oncol. 10, 249–255. 10.1001/jamaoncol.2023.5276 38153722

[B89] Willmore-Payne C. Holden J. A. Wittwer C. T. Layfield L. J. (2008). The use of EGFR exon 19 and 21 unlabeled DNA probes to screen for activating mutations in non-small cell lung cancer. J. Biomol. Tech. 19, 217–224. 19137110 PMC2563920

[B90] Wong J. C. Bathina M. Fiscus R. R. (2012). Cyclic GMP/protein kinase G type-Iα (PKG-Iα) signaling pathway promotes CREB phosphorylation and maintains higher c-IAP1, livin, survivin, and Mcl-1 expression and the inhibition of PKG-Iα kinase activity synergizes with cisplatin in non-small cell lung cancer cells. J. Cell Biochem. 113, 3587–3598. 10.1002/jcb.24237 22740515

[B91] Xiang G. Yang L. Qin J. Wang S. Zhang Y. Yang S. (2024). Revealing the potential bioactive components and mechanism of Qianhua Gout Capsules in the treatment of gouty arthritis through network pharmacology, molecular docking and pharmacodynamic study strategies. Heliyon 10, e30983. 10.1016/j.heliyon.2024.e30983 38770346 PMC11103544

[B92] Xiao L. Lan X. Shi X. Zhao K. Wang D. Wang X. (2017). Cytoplasmic RAP1 mediates cisplatin resistance of non-small cell lung cancer. Cell Death Dis. 8, e2803. 10.1038/cddis.2017.210 28518145 PMC5520727

[B93] Yang D. Feng W. Zhuang Y. Liu J. Feng Z. Xu T. (2021). Long non-coding RNA linc00665 inhibits CDKN1C expression by binding to EZH2 and affects cisplatin sensitivity of NSCLC cells. Mol. Ther. Nucleic Acids 23, 1053–1065. 10.1016/j.omtn.2021.01.013 33664990 PMC7887328

[B94] Yang X. Liu Q. Li G. (2024). Anti-NSCLC role of SCN4B by negative regulation of the cGMP-PKG pathway: Integrated utilization of bioinformatics analysis and *in vitro* assay validation. Drug Dev. Res. 85, e22192. 10.1002/ddr.22192 38678552

[B95] Yoneda K. Imanishi N. Ichiki Y. Tanaka F. (2019). Treatment of non-small cell lung cancer with EGFR-mutations. J. uoeh 41, 153–163. 10.7888/juoeh.41.153 31292359

[B96] Yousaf M. Hammond N. L. Peng J. Wahyuono S. Mcintosh K. A. Charman W. N. (2004). New manzamine alkaloids from an Indo-Pacific sponge. Pharmacokinetics, oral availability, and the significant activity of several manzamines against HIV-I, AIDS opportunistic infections, and inflammatory diseases. J. Med. Chem. 47, 3512–3517. 10.1021/jm030475b 15214779 PMC4928483

[B97] Yu L. Yang R. Long Z. Tao Q. Liu B. (2024). Targeted therapy of non-small cell lung cancer: mechanisms and clinical trials. Front. Oncol. 14, 1451230. 10.3389/fonc.2024.1451230 39391239 PMC11464343

[B98] Zhang Q. Wang R. Xu L. (2023). Clinical advances in EGFR-TKI combination therapy for EGFR-mutated NSCLC: a narrative review. Transl. Cancer Res. 12, 3764–3778. 10.21037/tcr-23-956 38192990 PMC10774042

[B99] Zhang T. Mo Z. Duan G. Tang R. Zhang F. Lu M. (2021). (125)I seed promotes apoptosis in non-small lung cancer cells via the p38 MAPK-MDM2-p53 signaling pathway. Front. Oncol. 11, 582511. 10.3389/fonc.2021.582511 33968713 PMC8096899

[B100] Zhao L. Zhang H. Li N. Chen J. Xu H. Wang Y. (2023). Network pharmacology, a promising approach to reveal the pharmacology mechanism of Chinese medicine formula. J. Ethnopharmacol. 309, 116306. 10.1016/j.jep.2023.116306 36858276

[B101] Zhao S. Qiu Z. He J. Li L. Li W. (2014). Insulin-like growth factor receptor 1 (IGF1R) expression and survival in non-small cell lung cancer patients: a meta-analysis. Int. J. Clin. Exp. Pathol. 7, 6694–6704. 25400749 PMC4230063

[B102] Zheng Y. He Z. Kong Y. Huang X. Zhu W. Liu Z. (2021). Combined metabolomics with transcriptomics reveals important serum biomarkers correlated with lung cancer proliferation through a calcium signaling pathway. J. Proteome Res. 20, 3444–3454. 10.1021/acs.jproteome.0c01019 34056907

[B103] Zhou C. Yu T. Zhu R. Lu J. Ouyang X. Zhang Z. (2023). Timosaponin AIII promotes non-small-cell lung cancer ferroptosis through targeting and facilitating HSP90 mediated GPX4 ubiquitination and degradation. Int. J. Biol. Sci. 19, 1471–1489. 10.7150/ijbs.77979 37056925 PMC10086754

[B104] Zhou Q. Hotta K. Deng Y. Yuan R. Quan S. Chen X. (2021). Advances in biosynthesis of natural products from marine microorganisms. Microorganisms 9, 2551. 10.3390/microorganisms9122551 34946152 PMC8706298

[B105] Zhu H. Zhang H. Jin F. Fang M. Huang M. Yang C. S. (2014). Elevated Orai1 expression mediates tumor-promoting intracellular Ca2+ oscillations in human esophageal squamous cell carcinoma. Oncotarget 5, 3455–3471. 10.18632/oncotarget.1903 24797725 PMC4116495

[B106] Zhu M. C. Zhang Y. H. Xiong P. Fan X. W. Li G. L. Zhu M. (2022). Circ-GSK3B up-regulates GSK3B to suppress the progression of lung adenocarcinoma. Cancer Gene Ther. 29, 1761–1772. 10.1038/s41417-022-00489-8 35821283

[B107] Zuehlke A. D. Beebe K. Neckers L. Prince T. (2015). Regulation and function of the human HSP90AA1 gene. Gene 570, 8–16. 10.1016/j.gene.2015.06.018 26071189 PMC4519370

